# Lipoprotein-Cholesterol Fractions in Marginalized Roma versus Majority Population

**DOI:** 10.3390/ijerph15010081

**Published:** 2018-01-06

**Authors:** Beáta Hubková, Gabriel Bódy, Jana Mašlanková, Anna Birková, Eugen Frišman, Vladimír Kraus, Mária Mareková

**Affiliations:** 1 Department of Medical and Clinical Biochemistry, Pavol Jozef Šafárik University in Košice Faculty of Medicine, Trieda SNP 1, 04011 Košice, Slovakia; jana.maslankova@upjs.sk (J.M.); anna.birkova@upjs.sk (A.B.); 2 Department of Medical and Clinical Biochemistry, Pavol Jozef Šafárik University in Košice Faculty of Medicine, Trieda SNP 1, 04011 Košice, Slovakia; present address: Medirex a.s., Magnezitárska 2/C, 04011 Košice, Slovakia; tappia1@gmail.com; 3 Department of Burns and Reconstructive Surgery, 1st Private Hospital Kosice-Saca, Lúčna 512/55, 04015 Košice-Šaca, Slovakia; efrisman@nemocnicasaca.sk; 4 Department of Gynaecology and Obstetrics, Louis Pasteur University Hospital, Rastislavova 785/43, 04001 Košice, Slovakia; vladimir.kraus@upjs.sk

**Keywords:** Roma people, lipoprotein, cholesterol, atherogenic lipoproteins

## Abstract

The trend of modern clinical biochemistry is to emphasize the composition and the quality of lipoproteins over their quantity. The serum lipoprotein fractions and subfractions were analyzed by the Lipoprint Lipoprotein Subfractions Testing System, the parameters of lipid profile, as total cholesterol (TC), low-density lipoprotein-cholesterol (LDL-C), high-density lipoprotein-cholesterol (HDL-C) and triacylglycerides (TAG) were determined by an automated selective biochemical analyzer. Our results showed a significantly lower concentration of cholesterol in the LDL fractions 1 and 2 and in the HDL fractions 8 to 10 in Roma compared to the majority population. The most significant differences between Roma and the majority population when considering body mass index (BMI), waist-to-hip ratio and the index of central obesity were in very low-density lipoproteins (VLDL), intermediate-density lipoproteins, fraction A (IDL-A) and LDL-2. The last two listed were significantly higher in the majority population. VLDL was significantly higher in overweight or obese Roma men and in Roma men with central obesity compared to men from the majority population, as well as in Roma women with normal weight and physiological waist-to-hip ratio compared to the women from majority population. Our study is among the first describing the distribution of lipoprotein subfractions in different ethnic groups.

## 1. Introduction

Lipoproteins are complex particles containing multiple proteins and lipids aimed at transporting fats throughout the organism. In addition to the doubtless importance of lipoproteins in fat absorption, utilization and cholesterol distribution, there is strong evidence that lipoproteins play substantial, but due to their different composition, varying roles in atherosclerosis. Nowadays, different methods are used for the fractionation of lipoproteins and for measuring cholesterol in the respective lipoprotein fractions and sub-fractions. These methods are based on continuous gradient ultracentrifugation, gradient or non-gradient gel electrophoresis, nuclear magnetic resonance and vertical analytical profiling. The idea of all these methods follows the first system of lipoprotein classification, designed by Fredrickson and Lees, who focused on the identification of primary biochemical defects of lipoprotein abnormalities [1]. They pointed out the necessity of determining the lipoprotein phenotype, since treatment of disorders vary with the different phenotypes. Over time, seven lipoprotein classes were identified based on their size and the lipid and apolipoprotein composition: chylomicrons, chylomicron remnants, very low-density lipoproteins (VLDL), intermediate-density lipoproteins (IDL), low-density lipoproteins (LDL), high-density lipoproteins (HDL) and an LDL-like particle with the specific apolipoprotein (a). Nowadays the concept of cardiac risk detection and prevention utilizing electrophoresis-based lipoprotein fractionation is even more developed. Recent non-gradient electrophoretic methods can separate up to seven LDL and ten HDL fractions, as well the VLDL and three IDL fractions, mainly based on their size. Small, dense LDL fractions 3 to 7, with an average particle size below 260 Å, appear to penetrate the endothelial barrier more easily than large fluffy LDL fractions 1 and 2. They are more likely to bind to proteoglycans, which leads to lipoprotein degradation by macrophage, thus initiating the cascade of events that results in atherosclerosis. The latest research suggests that differences in the distribution of lipoproteins may also have an ethnic basis [2].

The aim of the present work is to compare the results of the lipid profile parameters determined by a biochemical analyzer and anthropometric variables with the results of the distribution of cholesterol linked to the respective lipoprotein particles in Roma by the Lipoprint Lipoprotein Subfractions Testing System. In addition, a comparison of the result from Roma with data from the majority population is also provided. The goal was to try to determine what is behind the high risk of cardiovascular disease (CVD) among Roma, despite the higher ratio of Roma with physiological total cholesterol (TC) and low-density lipoprotein-cholesterol (LDL-C) values compared to the majority population, as indicated in our previous study [3].

## 2. Materials and Methods 

The analysis of the lipoprotein fractions and subfractions in fasting serum with a TC of over 2.59 mmol/L (100 mg/dL) was carried out on the Lipoprint Lipoprotein Subfractions Testing System (Quantimetrix, Redondo Beach, CA, USA). Principle of operation: lipoproteins are stained with a lipophilic dye and separated using a high resolution electrophoretic equipment on a linear polyacrylamide gel. The resolved bands of lipoprotein fractions and subfractions are scanned by digital scanner. The generated image is analyzed using Lipoware software (version 1.82, Quantimetrix, Redondo Beach, CA, USA) to calculate the cholesterol level for each of the fractions. Liposure control material was used to monitor accuracy and precision.

The parameters of lipid profile, as TC, LDL-C, high-density lipoprotein-cholesterol (HDL-C) and triacylglycerides (TAG) were determined using the selective biochemical analyzer ADVIA 2400 (Siemens Healthcare GmbH, Erlangen, Germany) in the accredited biochemical laboratory Medirex (Medirex Group, Košice, Slovakia).

## 3. Project Participants 

Probands (*n* = 166) were selected from adult participants of the cross-sectional population-based HepaMeta project [3] conducted in eastern Slovakia. In addition to monitoring the occurrence of viral hepatitis, the project was tasked with mapping the incidence of metabolic syndrome in the population living in separated and segregated Roma settlements compared to the majority population. In our study, 68 Roma (34 men and 34 women) aged 38 ± 9 years formed the study group, while the control group consisted of 98 non-Roma participants (49 men and 49 women) aged 36 ± 7 years. Study participants responded to a questionnaire developed by Roma health mediators and community workers, public health experts and academics. Affiliation to the Roma ethnic group was established based on replies to the questionnaire used.

The fasting blood samples were taken by trained medical personnel in the clinics of the cooperating general practitioners. The lipid profile of the respondents was mapped by determining the serum levels of TC, LDL-C, HDL-C and TAG and calculating the atherogenic index of plasma (AIP) combined with the determination of the fractions and subfractions of lipoproteins. The same personnel performed the anthropometric measurements on the participants: body weight and height, waist circumference (WC) and hip circumference (HC) using standardized equipment and procedures. The reference values of the serum lipid parameters are listed in Table 1.

Body mass index (BMI) was calculated as the body weight (BW) in kg divided by squared body height (BH^2^) in square meters:

BM = BW/BH^2^ (1)

The index of central obesity (ICO) was calculated as WC in centimeters divided by BH in centimeters:

ICO = WC/BH(2)

The waist-to-hip ratio (WHR) was calculated as WC in centimeters divided by HC in centimeters:

WHR = WC/HC(3)

AIP was calculated as logarithmically transformed ratio of molar concentrations of TAG to HDL-C:

AIP = log(TAG/HDL-C)(4)

Differences between group means were performed using a two-sample *t*-test, assuming equal variances. The strength of the linear relationship between the two variables was expressed by the Pearson correlation coefficient; a *p* value < 0.05 was estimated as being statistically significant. Means and standard deviations are reported in terms of the original distributions.

The study was approved by the Ethics Committee of the Faculty of Medicine, P. J. Šafárik University in Košice (No. 104/2011). Detailed information about our study and its procedures was given to all respondents, and informed consent was obtained prior to the medical examination.

## 4. Results

The participants were selected to be representative of people with physiological and pathological values of lipid profile. Whereas the HDL-C values of the study group determined by the biochemical analyzer were significantly lower compared to the control group (*p* = 0.046, Table 2), detailed fractionation of the lipoproteins was executed.

A lower concentration of the atherogenic HDL fractions 8 to 10 in the study group compared to the control group (*p* = 0.003, Table 3) was confirmed. Despite the statistically significant difference in the non-atherogenic LDL-C fractions 1 and 2 between the study group and the control group (*p* = 0.045, Table 3), no significant difference was detected in the total LDL-C between the mentioned groups (*p* = 0.368, Table 2).

Anthropometric variables: body weight and height, WC and HC, as well the subsequently determined data of WHR, are summarized for men and women separately due to different expected and/or cut-off values (Table 4). Roma men and women in the study group are significantly shorter in height (*p* < 0.001, both) and have significantly lower body weight (*p* = 0.030 and 0.006, for men and women, respectively) compared to the majority population in the control group. The average values of WHR in Roma men and Roma women, but not in participants from the majority population, were above the cut-off values. Nevertheless, no significant differences in BMI or WHR values (the most widely used determinants of obesity) between the study group and the control group were recorded.

Moreover, the index of central obesity in women was significantly higher in the study group compared to the control group (*p* = 0.011, Table 4). Both BMI and WC correlated significantly with TAG and HDL-C in the whole group. The inappropriateness of BMI as a marker of obesity was reported in Roma women, as no significant correlation was detected between BMI and either TC or LDL-C. Despite the pathological average values of ICO in Roma (0.6 ± 0.1 both in men and women, Table 4), significant correlation was detected between ICO and the lipid profile parameters in Roma men, but not in Roma women (Table 5).

**Figure 1:**
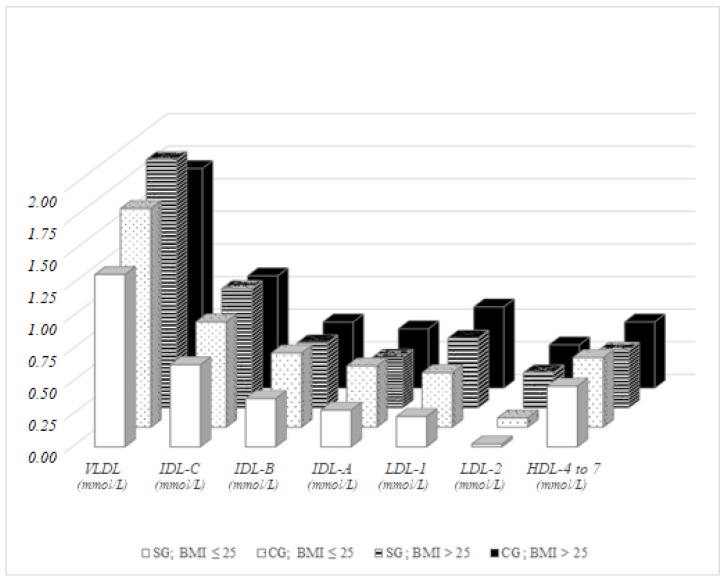
Lipoprotein fractions in men, BMI-dependent distribution.

Furthermore, we focused on the impact of selected indicators of obesity (BMI, WHR and ICO) with the cholesterol in the lipoprotein fractions determined by Lipoprint assay. In men of the control group with a physiological BMI (BMI ≤ 25) significantly higher concentrations of VLDL (*p* = 0.050), IDL-C (*p* = 0.050), IDL-B, IDL-A (*p* = 0.005 for both), LDL-1 (*p* = 0.013) and LDL-2 (*p* = 0.017) were observed compared to men with physiological BMI in the study group. In men with pathological BMI values (BMI > 25), higher VLDL (*p* = 0.027) and significantly lower concentrations of intermediate HDL-4–7 (*p* = 0.013) were observed in the study group. By observation of the differences between men with physiological and pathological values of BMI, six significantly different lipoprotein fractions were found in the study group (VLDL, IDL-C, IDL-B, IDL-A, LDL-1 and LDL-2, *p* = 0.001, *p* = 0.002, *p* = 0.011, *p* = 0.010, *p* < 0.001 and *p* = 0.001, respectively) and two in the control group (LDL-1 and LDL-2, *p* = 0.005 and *p* < 0.001, respectively). Concentrations of VLDL did not vary significantly in the control group men based on their BMI (Figure 1).

**Figure 2:**
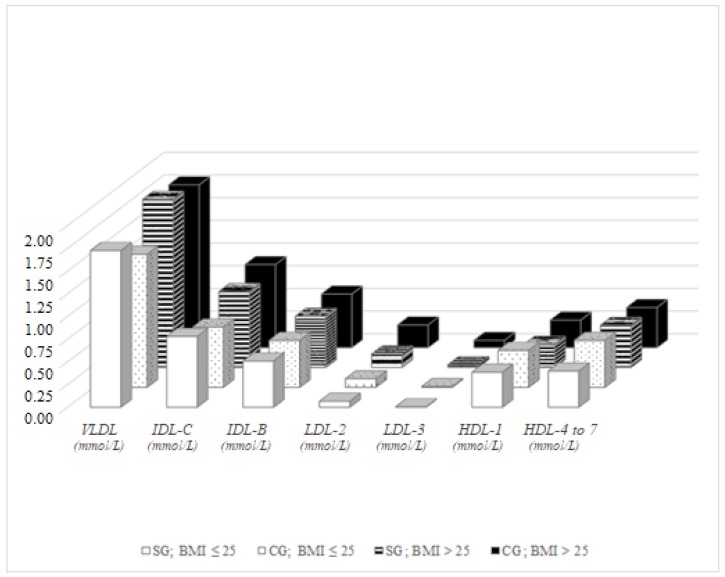
Lipoprotein fractions in women, BMI-dependent distribution.

In women with physiological BMI values, significant differences between the study group and control group were detected in VLDL (*p* = 0.049) and in intermediate HDL fractions 4–7 (*p* = 0.009). While in men VLDL levels were higher in the control group compared to the study group, in study group women significantly higher values of VLDL were observed compared to the majority population. No significant differences were detected between women with pathological BMI values between the study and the control group, except LDL-3 values, which were significantly higher in the control group compared to the study group (*p* = 0.042). Significant differences in lipoprotein fractions were not detected even among Roma women with physiological and pathological BMI values, whereas in the control group significant differences were observed in VLDL (*p* = 0.002), IDL-C (*p* = 0.001), IDL-B (*p* = 0.035), non-atherogenic LDL-2 (*p* = 0.007), atherogenic LDL-3 (*p* = 0.006) and intermediate HDL-4–7 (*p* = 0.027). VLDL was higher in control group women with pathological BMI values, which is in accordance with the elevated LDL-3 and decreased non-atherogenic HDL fractions (Figure 2).

**Figure 3:**
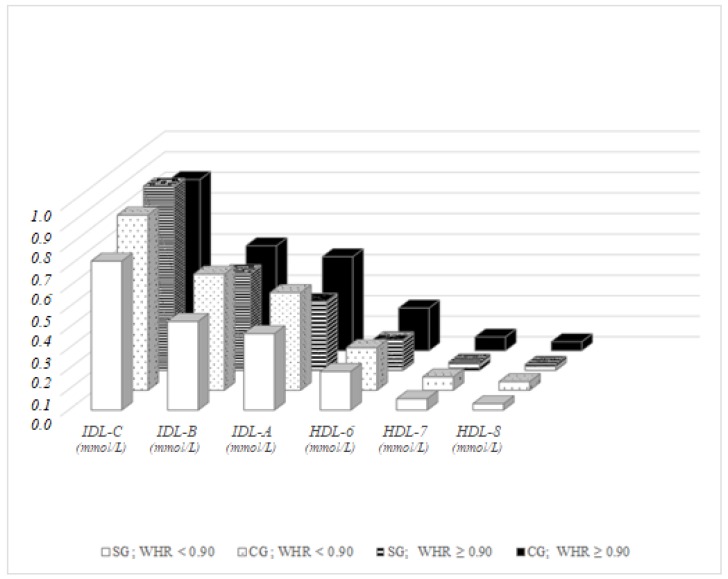
Lipoprotein fractions in men, distribution dependent on WHR.

Differences in lipoprotein fractions in men with physiological values of WHR (WHR < 0.90) were observed in IDL-B (*p* = 0.024) and IDL-A (*p* = 0.039), with significantly higher values in the control group compared to the study group, similar to the BMI-dependent results. In men with pathological WHR, the concentration of cholesterol in fractions IDL-A (*p* = 0.014), HDL-6 (*p* = 0.002), HDL-7 (*p* = 0.001) and HDL-8 (*p* = 0.008) were different between the representatives of the study group and the control group. No significant difference was recorded between men with physiological and pathological WHR in the fractions, either in the study group or in the control group, except in the concentrations of IDL-C in the study group, which was significantly higher in Roma men with pathological values of WHR (*p* = 0.036) compared to those with physiological WHR (Figure 3).

**Figure 4:**
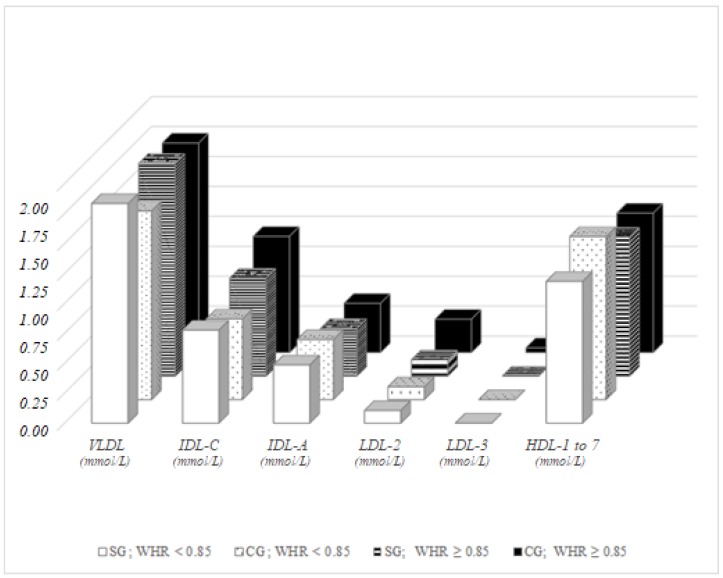
Lipoprotein fractions in women, distribution dependent on WHR.

Based on this finding, WHR in men does not appear to be a good parameter reliably indicating a predisposition to cardiovascular disease. Roma women with physiological WHR (WHR < 0.85) possessed significantly higher VLDL (*p* = 0.045) and significantly lower HDL-1–7 fractions (*p* = 0.016) compared to women with physiological WHR in the control group. Comparing to women with pathological WHR, majority women had significantly higher levels of IDL-C (*p* = 0.030), LDL-2 (*p* = 0.015) and LDL-3 (*p* = 0.026). While no significant differences were observed in lipoprotein fractions among Roma women classified based on WHR, women in the control group with pathological WHR had significantly higher IDL-C (*p* < 0.001), LDL-2 (*p* = 0.003) and LDL-3 (*p* = 0.007) compared to the control group women with physiological WHR (Figure 4).

**Figure 5:**
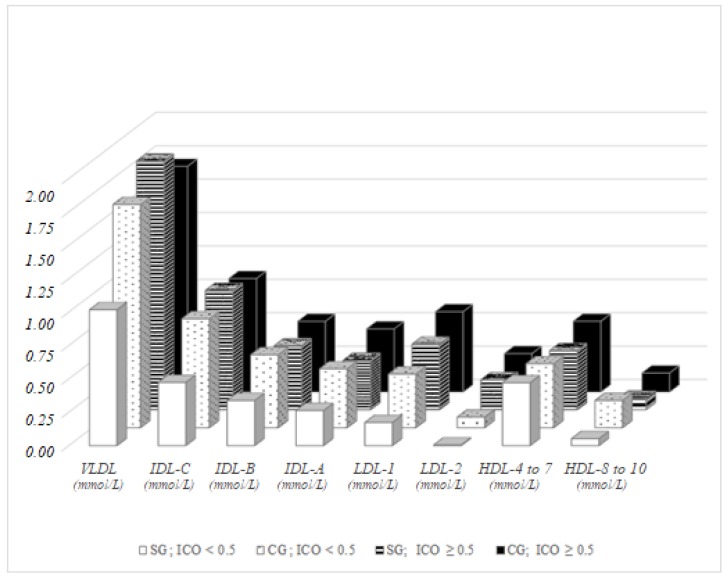
Lipoprotein fractions in men, distribution dependent on ICO.

Significant differences were observed between men in the control group and men in the study group with physiological ICO (ICO < 0.5), with a higher proportion of the fractions in majority group, namely in VLDL (*p* = 0.015), IDL-C (*p* = 0.008), IDL-B (*p* = 0.040), IDL-A (*p* = 0.047), LDL-1 (*p* = 0.030) and LDL-2 (*p* = 0.045). In men with pathological ICO, significant differences were recorded between representatives of the study group and the control group in IDL-A (*p* = 0.008), LDL-1 (*p* = 0.042), intermediate HDL-4–7 (*p* = 0.002) and atherogenic small HDL-8–10 (*p* = 0.024). A significant difference in the control group divided based on ICO was observed in just two parameters: LDL-1 (*p* = 0.013) and LDL-2 (*p* = 0.008); but in six parameters in the study group: VLDL and IDL-C (*p* < 0.001 both), IDL-B (*p* = 0.018), IDL-A (*p* = 0.026), LDL-1 (*p* = 0.001) and LDL-2 (*p* = 0.013, Figure 5).

**Figure 6:**
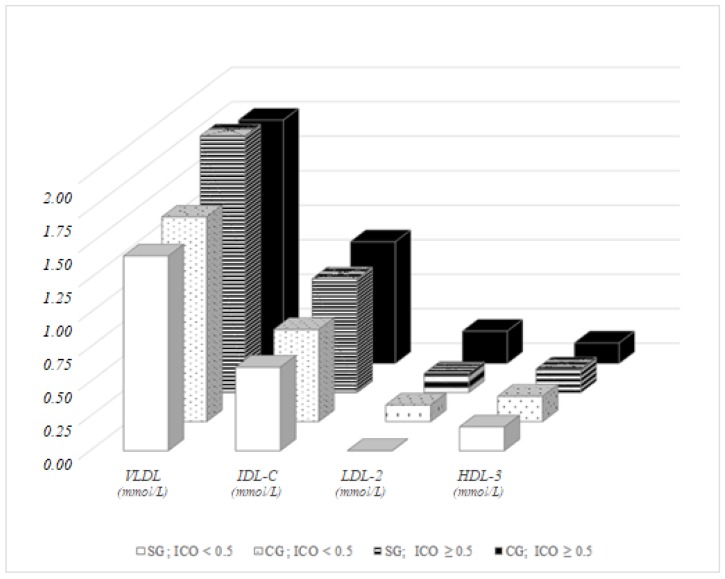
Lipoprotein fractions in women, distribution dependent on ICO.

In women with pathological ICO, no significant differences were recorded between representatives of the study group and the control group, except in the concentration of LDL-2 (*p* = 0.041). The difference in VLDL between women based on the ICO parameter was more pronounced in the control group (*p* = 0.007) compared to the study group (*p* = 0.041), and both were statistically significant. An ICO-dependent comparison pointed to significant differences in IDL-C, LDL-2 and HDL-3 in the control group (*p* = 0.003, 0.040, and 0.031, respectively) and in LDL-2 in the study group (*p* = 0.038). While IDL-C and LDL-2 were higher in pathological values of ICO, HDL-3 showed a modest but opposite trend (Figure 6).

## 5. Discussion

For many years, regardless of HDL and LDL sub-fractions, it was believed that the cholesterol transported in HDL was “good”, while the other included in LDL was “bad”. Nowadays, recent research has changed this opinion, pointing to the importance of both HDL and LDL subfractions and focusing more on their composition and metabolisms. The emerging opinion is that the quality of HDL and LDL particles may be more important than their quantity. Several studies have clearly shown that individuals with predominantly small dense LDL particles, along with raised TAG and decreased HDL, are at increased atherosclerotic risk, even if their LDL-cholesterol concentration is not increased [4]. Ethnic and racial differences in risk factors, atherosclerosis and cardiovascular disease have also been reported [5,6].

The poor social and economic conditions of Roma living in settlements largely contribute to the occurrence of various metabolic disorders as well as a higher risk of CVD [7]. The life expectancy of Roma is around 12 years less than that of the majority population [8]. Based on the data collected in the HepaMeta project, 27.6% of examined Roma females and 26.9% of males are overweight [9]. They are more likely to smoke on a daily basis and are heavier smokers compared to the majority population. Nevertheless, in the same HepaMeta project, there are more Roma with physiological TC and LDL-C level compared to the majority population [3], whilst precisely elevated TC and LDL-C concentrations are associated with atherosclerosis and a subsequent risk of CVD [10].

This study focused on finding differences in the distribution of cholesterol in lipoprotein subfractions, since average values of TC and LDL-C in Roma are more convenient when compared to majority population of eastern Slovakia [3], although they do not correlate with a higher risk of CVD in Roma [11]. In our study, the participants were selected to be representative of people with physiological and pathological values of the lipid profile both in the study group (Roma) as well in the control group (the majority population of eastern Slovakia). To optimize the predictive capacity of the lipid profile, some atherogenic indexes were defined, such as the AIP, WHR and ICO.

The major findings of this study are: at comparable average values of LDL-C concentrations among Roma and the majority population, there was a significantly lower concentration of cholesterol in the non-atherogenic low-density lipoprotein fractions 1 and 2 in Roma compared to the majority population. The statistically significant lower concentration of HDL-C in Roma compared to the majority population was caused mostly by the low concentration of cholesterol in the atherogenic high-density lipoprotein fractions 8–10. Significantly different values of body weight and height between Roma and the majority population were not reflected in the average anthropometric/atherogenic BMI and WHR indexes, whilst Roma had slightly worse results, compared to the majority population.

The average value of the ICO index was pathological both in Roma men and women, but not in the majority population, and this difference was substantial in men and statistically significant in women.

The major advantage of the selected anthropometric variables—BMI, WHR and ICO—is their use in the evaluation of obesity and subsequent cardiovascular risk. Moreover, ICO is applicable in subjects who are shorter than the general population, and with its one common cut-off value, it is applicable for evaluation of central obesity across races and genders [12]. Our study confirmed the suitability of this index in Roma, as it pointed out many subjects with central obesity, even though they had physiological BMI. The categorization of the probands according physiological and pathological values of the selected anthropometric variables should serve to point out differences in lipoprotein fraction between Roma and the majority population. Differences in subjects with pathological values were expected; the emphasis was on finding a disparity in subjects with a physiological anthropometric variable. WHR was revealed to be the least reliable parameter in defining atherogenity, since a difference in lipoprotein distribution was found mostly in subjects with pathological values and less often between subjects with physiological values.

While recent studies favor ICO over BMI, our findings pointed to similarities in lipoprotein distribution in men when categorizing subjects based on ICO and BMI.

As is well known, increased VLDL and LDL are often associated with obesity. Representatives of majority men from eastern Slovakia with physiological values of ICO and BMI possessed higher values of VLDL, fractions of IDL, large LDL-1 and LDL-2 compared to the Roma. Therefore, in men of normal weight in the control group, greater cardiovascular risk in comparison to Roma men of normal weight is assumed. We consider the VLDL and the IDL particles in particular as contributing to the development of atherosclerosis, surprisingly not in Roma but in the male majority population without visible signs of overweight or obesity. On the other hand, when comparing subjects with pathological BMI or ICO, higher values of VLDL but lower LDL-1 and LDL-2 were detected in Roma men compared to the majority, which should be the result of an ethnic variation in VLDL metabolism. A significant difference was observed in the intermediate fractions HDL-6, HDL-7, as well in small HDL-8, with lower concentrations in Roma men, which is in accordance with our previous study [3] that highlighted the alarming low levels of HDL-C in Roma.

Although the average value of ICO in Roma women was pathological, just moderate differences in lipoprotein particle distribution were detected when comparing Roma women with or without central obesity. More differences were detected when comparing Roma women and majority women with physiological BMI values, mostly in intermediate HDL-5 and HDL-6 particles. This should be explained by the fact that BMI is not the most reliable variable in evaluating obesity among Roma, who are significantly shorter in height compared to the Slovak majority.

Our study is among the first describing the distribution of lipoprotein subfractions in different ethnic groups. The observed differences between Roma and the majority when considering the anthropometric variables BMI, WHR and ICO were mostly in the VLDL, IDL-A and LDL-2 fractions.

Gradient gel electrophoresis is a widely accepted method for measuring lipoprotein particle number and size. However, nuclear magnetic resonance (NMR) spectroscopy could provide additional important insights.

## 6. Conclusions

Evaluation of disease-risk based on purely basic biochemical measures or anthropometric variables is in today’s rapidly changing multi-ethnic world insufficient. Increased attention should be paid to the quality of the respective HDL and LDL fractions as well as to the increasing quantity of the VLDL, especially in evaluation of the health status of different ethnic groups. Since lipoprotein subclasses are promising predictors of coronary disease, additional studies are needed to investigate differences in the distribution of these subclasses among various populations with different genetic background and lifestyles.

## Figures and Tables

**Table 1:** Reference values of serum lipid parameters and anthropometric variables.

Variable	Reference Values
TC (mmol/L)	0–5.0
TAG (mmol/L)	0.45–1.7
HDL-C (mmol/L)	1.0–2.7 ^a^/1.4–2.7 ^b^
LDL-C (mmol/L)	<3.0/<2.5 *
sG (mmol/L)	4.5–5.5
AIP	<0.11
BMI (kg/m^2^)	18.5–25
ICO	<0.5
WC (cm)	<102 ^a^/88 ^b^
WHR	<0.90 ^a^/0.85 ^b^

^a^ in men; ^b^ in women; * at risk. TC, total cholesterol; TAG, triacylglycerides; HDL-C, high-density lipoprotein-cholesterol; LDL-C, low-density lipoprotein-cholesterol; sG, serum glucose; AIP, atherogenic index of plasma; BMI, body mass index; ICO, index of central obesity; WC, waist circumference; WHR, waist-to-hip ratio.

**Table 2:** Average values of lipid profile parameters.

Variable	Study Group	Control Group	*p*
TC (mmol/L)	5.7 ± 1.3	5.9 ± 1.1	0.096
TAG (mmol/L)	1.63 ± 1.01	1.73 ± 1.11	0.269
HDL-C (mmol/L)	1.2 ± 0.3	1.3 ± 0.4	0.046 *
LDL-C (mmol/L)	3.1 ± 0.9	3.2 ± 0.8	0.368
sG (mmol/L)	5.0 ± 1.4	5.0 ± 0.7	0.310
AIP	0.08 ± 0.32	0.09 ± 0.31	0.438

*p*, *p* value of a two-sample *t*-test assuming equal variances; * *p* < 0.050; TC, total cholesterol; TAG, triacylglycerides; HDL-C, high-density lipoprotein-cholesterol; LDL-C, low-density lipoprotein-cholesterol; sG, serum glucose; AIP, atherogenic index of plasma.

**Table 3:** Comparison of average values of cholesterol in lipoprotein fractions and LDL size.

Variable	Study Group	Control Group	*p*
VLDL (mmol/L)	1.76 ± 0.50	1.67 ± 0.43	0.098
IDL-C (mmol/L)	0.82 ± 0.28	0.82 ± 0.28	0.459
IDL-B (mmol/L)	0.50 ± 0.19	0.54 ± 0.17	0.054
IDL-A (mmol/L)	0.39 ± 0.18	0.46 ± 0.19	0.013 *
LDL size (Å)	273 ± 5	272 ± 5	0.104
LDL-1 and 2 (mmol/L)	0.59	0.71	0.045 *
LDL-3 to 7 (mmol/L)	0.04	0.08	0.056
HDL-1 to 7 (mmol/L)	1.09	1.14	0.185
HDL-8 to 10 (mmol/L)	0.08	0.12	0.003 **

For simplification, a summary of the non-atherogenic (LDL-1 and 2 and HDL-1 to 7) and atherogenic fractions (LDL-3–7 and HDL-8–10) of LDL-C and HDL-C are listed. *p*, *p* value of a two-sample *t*-test assuming equal variances; * *p* < 0.050 ** *p* < 0.010.

**Table 4:** Comparison of anthropometric variables, BMI, WHR and ICO of the participants.

Variable	Men			Women		
	Study Group *n* = 34	Control Group *n* = 49	*p*	Study Group *n* = 34	Control Group *n* = 49	*p*
Body weight (kg)	79 ± 17	86 ± 15	0.030 *	65 ± 13	72 ± 14	0.006 **
Height (cm)	168 ± 6	177 ± 7	0.000 ***	154 ± 7	166 ± 7	0.000 ***
BMI	28 ± 5	27 ± 5	0.322	27 ± 5	26 ± 5	0.183
WC (cm)	96 ± 15	96 ± 11	0.432	88 ± 11	88 ± 13	0.489
HC (cm)	104 ± 10	106 ± 7	0.176	103 ± 10	106 ± 10	0.108
WHR	0.91 ± 0.08	0.90 ± 0.07	0.301	0.86 ± 0.05	0.83 ± 0.07	0.057
ICO	0.6 ± 0.1	0.5 ± 0.1	0.064	0.6 ± 0.1	0.5 ± 0.1	0.011 **

*p*, *p* value of a two-sample *t*-test assuming equal variances; * *p* < 0.050; ** *p* < 0.010; *** *p* < 0.001; BMI, body mass index; WC, waist circumference; HC, hip circumference; WHR, waist-to-hip ratio; ICO, index of central obesity.

**Table 5:** Correlation between the anthropometric variables and the values of the lipid profile parameters.

			TC	*p*	TAG	*P*	HDL-C	*p*	LDL-C	*P*
Body weight	SG	m	0.543	0.001 **	0.652	0.000 ***	−0.649	0.000 ***	0.542	0.001 **
		w	0.090	0.614	0.545	0.001 **	−0.537	0.001 **	0.033	0.854
	CG	m	0.209	0.150	0.535	0.000 ***	−0.595	0.000 ***	0.158	0.277
		w	0.368	0.010 *	0.446	0.001 **	−0.398	0.005 **	0.432	0.002 **
Height	SG	m	−0.027	0.879	0.278	0.111	−0.236	0.180	0.040	0.822
		w	−0.180	0.317	0.102	0.574	−0.373	0.033 *	−0.175	0.331
	CG	m	−0.193	0.185	0.213	0.141	−0.332	0.020 *	−0.205	0.158
		w	0.191	0.193	0.186	0.205	−0.088	0.554	0.164	0.264
BMI	SG	m	0.596	0.000 ***	0.594	0.000 ***	−0.622	0.000 ***	0.577	0.000 ***
		w	0.169	0.340	0.530	0.001 **	−0.397	0.020 *	0.102	0.564
	CG	m	0.289	0.044 *	0.452	0.001 **	−0.491	0.000 ***	0.252	0.080
		w	0.305	0.033 *	0.403	0.004 **	−0.394	0.005 **	0.394	0.005 **
WC	SG	m	0.561	0.001 **	0.649	0.000 ***	−0.628	0.000 ***	0.611	0.000 ***
		w	0.286	0.107	0.544	0.001 **	−0.421	0.015 *	0.223	0.212
	CG	m	0.263	0.071	0.481	0.001 **	−0.518	0.000 ***	0.218	0.137
		w	0.341	0.017 *	0.433	0.002 **	−0.333	0.019 *	0.389	0.006 **
HC	SG	m	0.616	0.000 ***	0.623	0.000 ***	−0.618	0.000 ***	0.598	0.000 ***
		w	0.294	0.096	0.458	0.007 **	−0.382	0.028 *	0.261	0.143
	CG	m	0.254	0.081	0.423	0.003 **	−0.546	0.000 ***	0.244	0.095
		w	0.171	0.240	0.172	0.238	−0.358	0.012 *	0.315	0.027 *
WHR	SG	m	0.375	0.029 *	0.500	0.003 **	−0.460	0.006 **	0.492	0.003 **
		w	0.144	0.424	0.392	0.024 *	−0.272	0.125	0.071	0.695
	CG	m	0.206	0.159	0.390	0.006 **	−0.349	0.015 *	0.145	0.327
		w	0.420	0.003 **	0.532	0.000 ***	−0.141	0.334	0.337	0.018 *
ICO	SG	m	0.593	0.000 ***	0.603	0.000 ***	−0.609	0.000 ***	0.630	0.000 ***
		w	0.083	0.640	0.312	0.073	−0.353	0.041 *	0.072	0.687
	CG	m	0.294	0.041 *	0.332	0.020 *	−0.308	0.031 *	0.261	0.070
		w	0.022	0.879	0.227	0.117	−0.353	0.013 *	0.122	0.405

*p*, *p* value of the two-tailed Pearson correlation coefficient; * *p* < 0.050; ** *p* < 0.010; *** *p* < 0.001; SG, study group; CG, control group; m, men; w, women.

## Abbreviations

AIP	atherogenic index of plasma
BMI	body mass index
BH	body height
BW	body weight
CG	control group
CVD	cardiovascular disease
HC	hip circumference
HDL	high-density lipoproteins
HDL-C	high-density lipoprotein-cholesterol
ICO	index of central obesity
IDL	intermediate-density lipoproteins
IDL-A	intermediate-density lipoproteins, fraction A
LDL	low-density lipoprotein
LDL-C	low-density lipoprotein-cholesterol
sG	serum glucose
SG	study group
TAG	triacylglycerides
TC	total cholesterol
VLDL	very low-density lipoproteins
WC	waist circumference in centimeters
WHR	waist-to-hip ratio

## Collaborators of the HepaMeta Team

Peter Jarčuška, Andrea Madarasová Gecková, Mária Mareková, Daniel Pella, Leonard Siegfried, Pavol Jarčuška, Lýdia Pastvová, Ján Fedačko, Jana Kollárová, Peter Kolarčik, Daniela Bobáková, Zuzana Veselská, Ingrid Babinská, Sylvia Dražilová, Jaroslav Rosenberger, Ivan Schréter, Pavol Kristian, Eduard Veselíny, Martin Janičko, Ladislav Virág, Anna Birková, Marta Kmeťová, Monika Halánová, Darina Petrášová, Katarína Cáriková, Viera Lovayová, Lucia Merkovská, Lucia Jedličková, and Ivana Valková.

## Abbreviations

AIP	atherogenic index of plasma
BMI	body mass index
BH	body height
BW	body weight
CG	control group
CVD	cardiovascular disease
HC	hip circumference
HDL	high-density lipoproteins
HDL-C	high-density lipoprotein-cholesterol
ICO	index of central obesity
IDL	intermediate-density lipoproteins
IDL-A	intermediate-density lipoproteins, fraction A
LDL	low-density lipoprotein
LDL-C	low-density lipoprotein-cholesterol
sG	serum glucose
SG	study group
TAG	triacylglycerides
TC	total cholesterol
VLDL	very low-density lipoproteins
WC	waist circumference in centimeters
WHR	waist-to-hip ratio
